# Integrating Extracellular Matrix Dynamics and Membrane-Associated Signaling: The Role of Clusterin-LRP1 Network in Cancer Cell Migration and Tumor Progression

**DOI:** 10.3390/cancers18111791

**Published:** 2026-05-30

**Authors:** Alessia Ciringione, Federica Rizzi, Sylvia Mangani, Zoi Piperigkou, Nikos Karamanos

**Affiliations:** 1 Laboratory of Biochemistry, Molecular Biology and Oncometabolism, Department of Medicine and Surgery, University of Parma, Via Volturno 39, 43125 Parma, Italy; alessia.ciringione@unipr.it; 2 Biochemistry, Biochemical Analysis & Matrix Pathobiology Research Group, Department of Chemistry, University of Patras, 26504 Patras, Greece; sylvia.mangani@upatras.gr (S.M.); zoipip@upatras.gr (Z.P.)

**Keywords:** clusterin, low-density lipoprotein receptor-related protein 1, membrane type 1 matrix metalloproteinase, CD44, syndecans, tumor cell migration, extracellular matrix remodeling, cytoskeletal dynamics

## Abstract

Tumor cell migration relies on the integration of extracellular matrix (ECM) remodeling, cell surface signaling regulating cytoskeleton dynamics, and epithelial-to-mesenchymal transition (EMT). Clusterin (CLU), a secreted glycoprotein, is involved in extracellular proteostasis and is known to interact with members of the LDL receptor family, including low-density lipoprotein receptor-related protein 1 (LRP1). Beyond its canonical chaperone activity, CLU is involved in several biological processes, including cell survival, apoptosis, tissue remodeling, inflammation and cancer progression. On the other hand, the membrane type 1 matrix metalloproteinase (MT1-MMP), functionally linked to CD44 and LRP1, represents a key membrane-associated molecule that may control cell adhesion and receptor-mediated uptake of ECM ligands and proteases. In this article, we critically highlight a hypothetical model in which secreted CLU (sCLU) may function as the central player of a dynamic membrane-associated network integrating proteolysis, endocytosis, and intracellular signaling. Based on recent literature findings and STRING analyses, LRP1, MT1-MMP, CD44, and cell surface matrix components, such as proteoglycans (PGs) and integrins, are likely to be involved. By coordinating this membrane-associated molecular crosstalk, sCLU may integrate ECM remodeling with cytoskeletal dynamics and EMT-related programs related to invasive behavior. Overall, this framework highlights a potential mechanism through which sCLU may contribute to tumor cell plasticity and aggressiveness, suggesting new avenues for therapeutic intervention.

## Simple Summary

Tumor progression and metastasis depend on a dynamic and reciprocal interaction between cancer cells and their surrounding environment, yet the mechanisms by which these complex processes are coordinated at the cell surface are not fully understood. Clusterin, a protein secreted outside cells, may play key roles in integrating these events, helping the assembly of key molecules on the cell membrane into a functional network that links extracellular matrix remodeling, endocytosis, and intracellular signaling. Through this organization, clusterin may promote changes in cell shape and behavior that promote cancer cell migration and invasion. By proposing this model, we aim to provide a new perspective on how these events are regulated and to highlight potential targets for future therapeutic strategies.

## 1. Introduction

Tumor cell migration represents a complex and tightly regulated process involving ECM remodeling, the reprogramming of signaling pathways controlling cytoskeletal dynamics, and EMT [1]. These mechanisms converge at the ECM–cell interface, in which the ECM represents not merely a natural structural bioscaffold, but it also actively modulates the availability of biochemical and mechanical cues. At the same time, the cell membrane functions as a complementary regulatory hub where these inputs are integrated through tightly controlled processes such as proteolysis, receptor trafficking, and endocytosis. Within this framework, cell–cell and cell–ECM interactions further contribute to shaping the spatial and temporal organization of signaling events, ultimately influencing cell adhesion, migration, and invasion [2].

Specific ECM components as well as membrane-associated proteins and enzymes play key roles in coordinating these events within the tumor microenvironment (TME). Among these, MT1-MMP (also known as MMP14) drives tumor migration through its ability to degrade ECM components, such as fibronectin and vitronectin, activate pro-MMP2, and promote the shedding of multiple surface proteins including LRP1, CD44, and heparan sulfate PGs (HSPGs). In addition, its cytoplasmic tail supports RhoA activation, which in turn contributes to modulating the cytoskeletal rearrangement through actin polymerization [3].

In this context, LRP1, as a type I transmembrane receptor, promotes the internalization of extracellular ligands as well as the turnover of cell membrane receptors. In this way LRP1 contributes to the regulation of proteolytic processes and ECM remodeling in the pericellular environment [4]. Notably, LRP1 promotes the endocytosis of adhesion receptors such as CD44, regulating their surface availability and contributing to the dynamic balance between cell adhesion and detachment required for efficient migration [5]. Furthermore, LRP1 acts as a signaling co-receptor by forming complexes with growth factors receptors, thereby linking membrane trafficking to intracellular signaling cascades associated with invasive behavior. In addition, its cytoplasmic tail engages adaptor and scaffold proteins involved in cytoskeletal attachment and dynamics [6].

During cancer dissemination, CD44, a major hyaluronan receptor, interacts with MMPs and stabilizes their localization to the leading edge of migrating cells, promoting spatially restricted ECM degradation. Specifically, CD44 functions as a scaffold for the assembly of MMPs with their substrates, including ECM components such as collagen, fibronectin, and laminin, as well as cell surface receptors, including CD44 itself. The interdependent relationship between MT1-MMP and CD44 highlights a functional interplay between cell adhesion and proteolytic remodeling [7]. Beyond its role in cell motility and pro-tumorigenic signaling, CD44 is a well-established cancer stem cell biomarker, strongly linked to tumor initiation, self-renewal, and resistance to therapy [8,9].

HSPGs are glycoproteins bearing covalently attached heparan sulfate chains and are mainly represented by membrane-associated PGs, such as syndecans (SDCs) and glypicans (GPCs), as well as pericellular PGs, including perlecan. Of note, HSPGs exert pleiotropic roles in cancer progression, importantly influencing cancer cell signaling and proliferation, migration, invasion, cell–matrix adhesion, and metastatic potential [10,11]. In particular, SDCs (SDC1-4) act as co-receptors for growth factors and chemokines, as well as interacting with matrix structural molecules and integrins, thus triggering intracellular pro-oncogenic signaling pathways [12,13]. Moreover, proteolytic shedding of SDC ectodomains by enzymes (i.e., MMPs) generates soluble SDCs (i.e., shed SDCs), which can act in both autocrine and paracrine manners to promote the crosstalk between cancer cells and the TME, hence driving metastasis [13,14]. Similarly, GPC1-6 function as co-receptors for a wide range of cell surface receptors and mediate cell–matrix interactions, thereby participating in multiple pathways involved in the regulation of cell fate and tumor progression [15]. Among pericellular HSPGs, perlecan contributes to ECM and basement membrane organization, regulates cell adhesion, sequesters growth factors, and modulates integrin-mediated signaling involved in cancer progression [16].

Other key extracellular molecules may further affect the activity of membrane-associated systems involved in tumor cell migration. Among these, secreted clusterin (sCLU), which is the first identified and the most prominent mammalian extracellular chaperone contributes to cellular homeostasis through three main activities: proteostasis maintenance, cytoprotection, and inflammation inhibition. Since its initial characterization, sCLU has been shown to promote protein clustering and aggregation [17,18]. Indeed, it acts as an ATP-independent chaperone that binds to misfolded proteins and facilitates their clearance through interactions with scavenger receptors, including members of the LRP family and HSPGs [19,20,21]. Furthermore, sCLU binds to membrane receptors and modulates intracellular signaling pathways involved in cell survival, stress responses, and cell migration [22,23].

The main components that contribute to this membrane-associated network, their localization and biological functions are summarized in Table 1.

**Figure 1:**
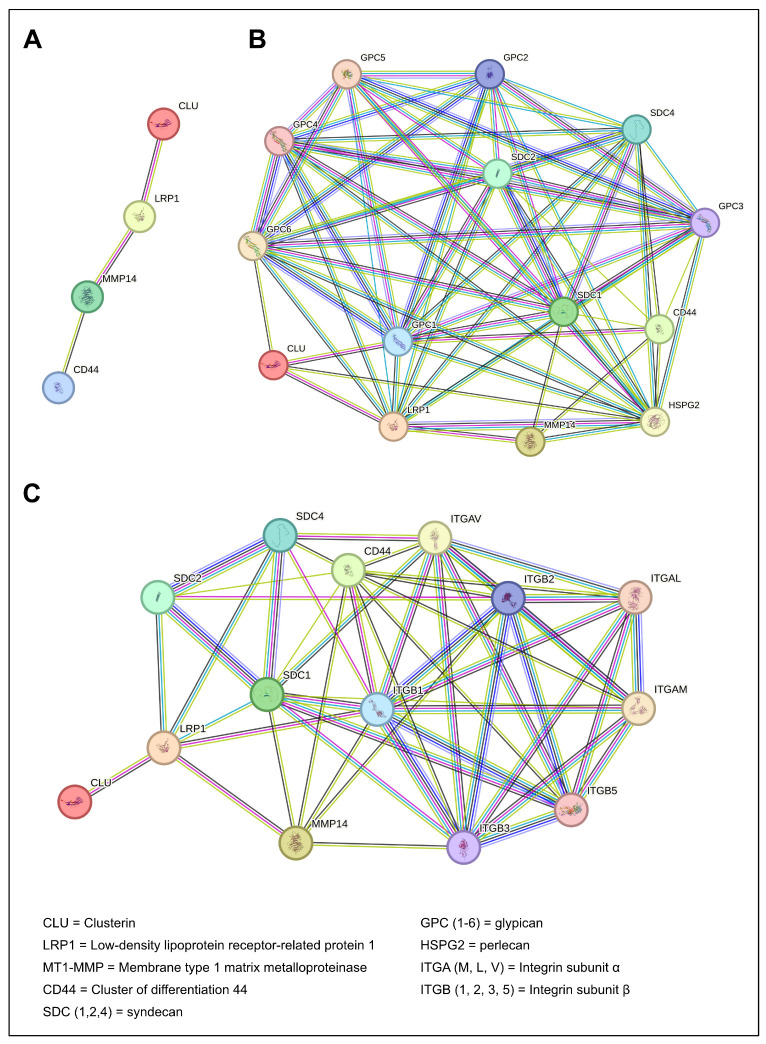
Protein–protein interaction (PPI) networks generated using the STRING database for the main players of the proposed signaling network. (**A**) PPI graph showing interactions among the main components of the proposed framework (CLU, LRP1, MT1-MMP, CD44). PPI graphs illustrating the interactions among the four main players and heparan sulfate proteoglycans (**B**), and syndecans and integrins (**C**). Network nodes represent proteins, whereas edges indicate predicted functional associations inferred from multiple evidence sources integrated within the STRING platform. Edge colors correspond to the type of supporting evidence as follows: pink, experimentally validated interactions; yellow, text mining evidence; black, co-expression evidence; blue, co-occurrence evidence; light blue, curated database interactions; light purple, protein homology. STRING database available at: https://string-db.org (version 12.0; accessed on 20 May 2026).

To further explore the potential relationships among these molecules, we performed a STRING-based protein–protein interaction (PPI) analysis including CLU, LRP1, MT1-MMP, CD44, HSPGs, and integrins, all implicated in ECM and cell membrane dynamics (Figure 1).

In this article, based on the available literature data and STRING-based network analyses, we highlight that sCLU, once secreted into the TME, may facilitate organization of a membrane-associated signaling network by promoting the interplay of LRP1, MT1-MMP, CD44, SDCs, GPCs, perlecan, and integrins. This is expected to increase the local density and spatial proximity of these components, thereby enhancing the efficiency and coordination of their functional interplay. This network may favor the coupling of membrane trafficking, proteolytic activity, and adhesion dynamics, ultimately accelerating receptor turnover while spatially restricting ECM remodeling. In parallel, these interactions may act as integrated signaling platforms that support intracellular pathways driving cytoskeletal reorganization, EMT reprogramming, and directional migration. Within this framework, sCLU may act not only as an extracellular chaperone but also as a potential upstream player of membrane dynamics, promoting a coordinated regulation of cell–ECM interactions and pro-tumorigenic cellular programs.

## 2. The ECM–Cell Interface in Tumor Cell Migration

The ECM macromolecular network is recognized as a key feature of TME, acting not only as a structural scaffold but also as a dynamic interface continuously shaped through bidirectional communication with tumor cells [28]. ECM is a highly organized three-dimensional network composed of diverse structural and functional macromolecules (including collagens, laminins, fibronectin, glycosaminoglycans/PGs, and glycoproteins, among others), as well as matrix-remodeling enzymes [29]. Moreover, the ECM integrates biochemical and mechanical signals that actively regulate cell adhesion, migration, and survival. On the other hand, tumor cells remodel the matrix by altering its composition, stiffness/remodeling, and architecture. These reciprocal interactions establish a self-sustaining feedback loop that drives tumor progression and metastatic dissemination, and thus contributes to therapy resistance [30].

A critical step in cancer development is the ability of tumor cells to overcome the physical constraints imposed by the basement membrane of the ECM to invade the surrounding stromal tissue [31]. In this context, membrane-anchored and secreted enzymes act as key mediators of pericellular proteolysis [32]. Specifically, MMPs consist of a large family of zinc-dependent, calcium-containing endopeptidases that are broadly upregulated across different cancer types [33]. Their primary function is to regulate ECM remodeling and intercellular communication through the proteolysis of both matrix substrates, such as collagens, various glycoproteins, and PGs, and non-matrix substrates, including growth factors and cytokines. Importantly, the shedding of these bioactive molecules can further amplify pro-tumorigenic pathways, thereby affecting multiple features of tumor progression, including cell survival and proliferation, migration, invasion, angiogenesis, immune evasion, and metastasis [3].

Among MMPs, MT1-MMP was the first proteinase to be identified on the surface of invasive cancer cells and has since been related to poor prognosis in multiple tumor types [34,35,36]. MT1-MMP exhibits a broad spectrum of substrates, including collagen types I, II, and III, fibronectin, vitronectin, and laminins. Furthermore, it can indirectly mediate the degradation of type IV collagen through the activation of pro-MMP2 [37]. Importantly, this process is regulated by tissue inhibitor of metalloproteinases 2 (TIMP2), which exerts a dual role in tumor cell migration. At low concentrations, TIMP2 promotes pro-MMP2 activation by bridging MT1-MMP and its substrate at the cell surface, thereby enhancing pericellular proteolysis and activating downstream signaling pathways, including the MAPK cascade. In contrast, at higher concentrations, TIMP2 inhibits MMP activity by blocking their catalytic sites, ultimately restraining cell migration and invasion [38,39].

Efficient localized ECM degradation depends on the polarized distribution of MT1-MMP at the leading edge of migrating cells. Indeed, MT1-MMP accumulates at specialized cell membrane protrusions, such as invadopodia, filopodia, and lamellipodia [40]. Its surface expression is dynamically controlled by endocytic and exocytic trafficking, allowing continuous recycling and targeted delivery to sites of invasion [41]. Notably, CD44 has been reported to drive MT1-MMP localization to lamellipodia through interaction with its hemopexin domain [42]. In turn, MT1-MMP cleaves the ectodomain of CD44 to promote the detachment of cells from the surrounding matrix [43]. This process is followed by intramembrane proteolysis that leads to the release of the CD44 intracellular domain, which can move to the nucleus where it modulates the transcription of genes involved in pro-tumorigenic programs [44].

In this framework, receptor-mediated trafficking has emerged as a critical regulator for coordinated proteolysis and signaling at the ECM–cell interface. The matricellular receptor LRP1, a member of the low-density lipoprotein receptor family, is involved in the modulation of multiple ECM-related pathways, as it interacts with a wide range of extracellular ligands as well as membrane-associated and intracellular proteins [45]. In particular, it mediates the endocytosis of several proteases, such as MMP2 and MMP9, either as free enzymes or in association with soluble partners, including the pro-MMP2:TIMP2 complex [46].

LRP1 also regulates the turnover of the urokinase-type plasminogen activator system. Specifically, it promotes the internalization of complexes formed by urokinase-type plasminogen activator (uPA), its receptor (uPAR), and plasminogen activator inhibitor-1 (PAI-1), thereby reducing uPAR levels at the cell surface [47]. Through this mechanism, LRP1 modulates plasmin generation, the activation of MMPs and the consequent degradation of ECM components. Notably, LRP1 has been shown to regulate the adhesion/deadhesion balance in tumor cells by promoting the internalization of CD44 [5].

Beyond its role in endocytosis, LRP1 also functions as a signaling co-receptor. For instance, it can bind to the platelet-derived growth factor receptor-β (PDGFR-β) forming a signaling complex that regulates cancer-related pathways, including the MAPK cascade and the PI3K/Akt signaling [48]. Furthermore, the cytoplasmatic tail of LRP1 interacts with a broad range of cytoplasmatic adaptors and scaffold proteins, including a close homologue of the cytoskeletal protein Talin (TLN) and OMP25, involved in cytoskeletal attachment and cell adhesion [6]. Interestingly, MT1-MMP induces LRP1 shedding at the cell surface of malignant cells [49], feeding a dynamic feedback loop between proteolysis and membrane trafficking. Soluble LRP1 fragments have been reported to retain biological activity by acting as “decoys” that bind extracellular ligands and modulate their availability, while also contributing to the activation of intracellular signaling pathways [50].

**Figure 2:**
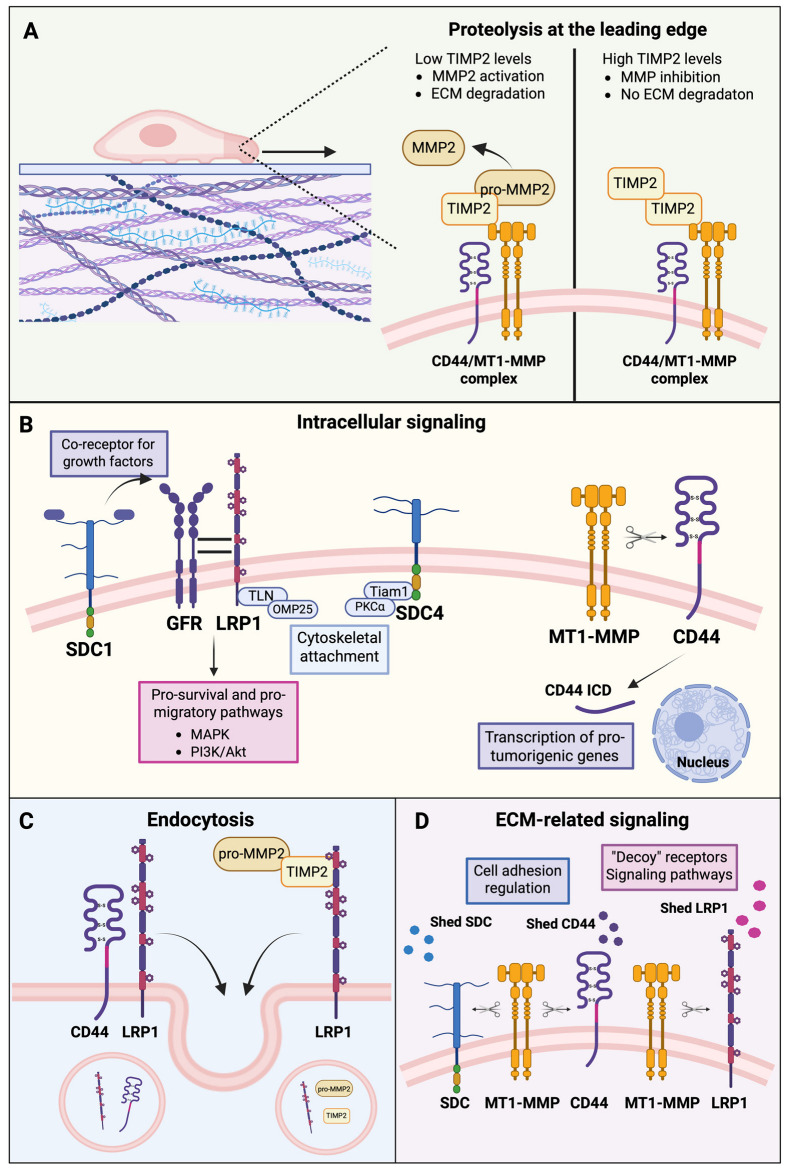
Integrated membrane networks coordinating endocytosis, proteolysis, and signaling during tumor cell migration. Tumor cell migration relies on the coordinated interplay between ECM remodeling, membrane receptor dynamics, and intracellular signaling pathways. (**A**) At the leading edge of the migrating cell, MT1-MMP binds to CD44, promoting localized ECM degradation and activation of pro-MMP2 in a TIMP2-dependent manner, thereby facilitating invasion. (**B**) Membrane-associated complexes involving LRP1, growth factor receptors (GFRs), and syndecans (SDC1 and SDC4), contribute to signal integration at the cell surface. In this context, SDCs can function as a co-receptor for growth factors, while SDC4 is also involved in the regulation of cytoskeletal organization and adhesion dynamics through PKCα- and Rho GTPase-dependent pathways. These membrane platforms also activate pro-survival and pro-migratory signaling cascades, including MAPK and PI3K/Akt pathways. In parallel, CD44 can undergo proteolytic cleavage by MT1-MMP, releasing its intracellular domain (CD44 ICD), which is translocated to the nucleus and promotes the transcription of pro-tumorigenic genes. (**C**) LRP1-mediated endocytosis regulates the internalization and recycling of membrane-associated proteins, including proteases and adhesion receptors, contributing to the spatial regulation of proteolysis and receptor availability. (**D**) Shedding of SDCs, CD44, and LRP1 releases soluble fragments that further modulate the extracellular and pericellular landscape. Created in BioRender. Piperigkou, Z. (2026) https://BioRender.com/hgrbi8x (accessed on 20 May 2026).

Collectively, these processes define a regulatory network at the ECM–cell interface, integrating proteolysis, receptor trafficking, and signaling to drive tumor cell migration (Figure 2).

## 3. The Role of sCLU in Cell Signaling and Tumor Progression

sCLU is a highly conserved, stress-responsive glycoprotein expressed in a wide range of tissues and biological fluids. The expression of sCLU is tightly and dynamically regulated at the transcriptional level, reflecting its role as a stress-responsive protein. Its gene expression is modulated by a complex interplay of signaling pathways and regulatory mechanisms, including hormones, growth factors, cytokines, and cellular stress responses such as the heat shock response and the unfolded protein response [51,52]. In addition, epigenetic mechanisms, such as DNA methylation, histone deacetylation, and microRNA-mediated regulation, contribute to fine-tuning CLU transcription in a context-dependent manner [53,54]. sCLU is synthesized through the canonical secretory pathway, beginning with translation of a precursor that includes an N-terminal signal peptide directing the protein into the endoplasmic reticulum (ER). There, the signal sequence is removed, disulfide bonds are formed, and the protein undergoes extensive glycosylation before being cleaved in the Golgi apparatus by a furin-like protease into α- and β-chains linked by disulfide bonds. The mature sCLU is then secreted as a heterodimer of two 40–45 kDa subunits. Under cellular stress, however, non-canonical intracellular CLU forms can arise due to altered processing, including failed ER translocation, retrotranslocation, alternative splicing, or alternative translation initiation [55]. Further work is required to better define the identity and function of these forms, as they have never been isolated under physiological conditions, nor have they been sequenced or crystallized [56].

**Figure 3:**
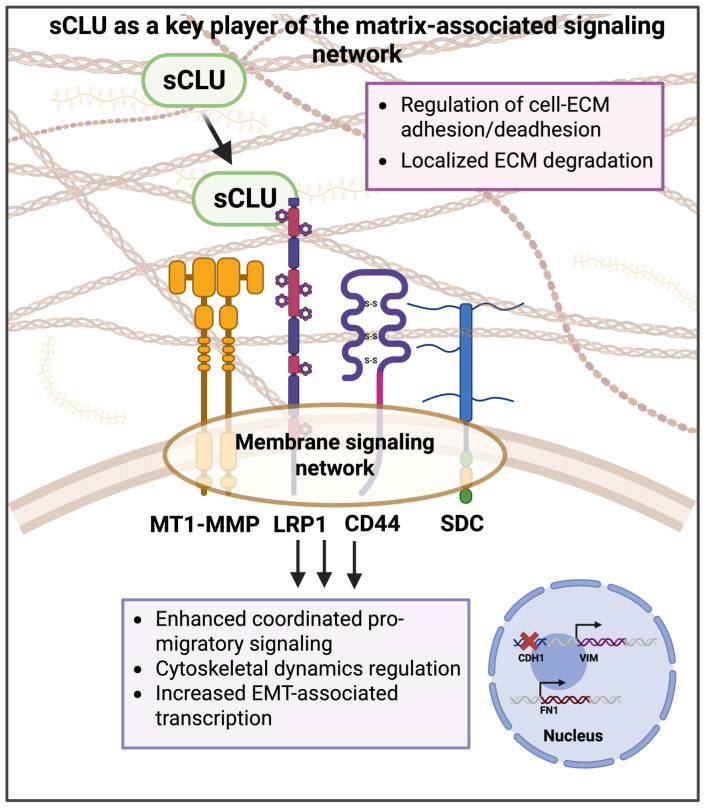
sCLU as a key player of the matrix-associated signaling network. sCLU promotes the formation of a membrane-associated signaling network by binding to LRP1 and facilitating the spatial co-localization of MT1-MMP, CD44 and SDCs at the cell surface. This organization enhances receptor cooperation and supports localized ECM degradation, dynamic regulation of cell–ECM adhesion, and activation of pro-migratory signaling pathways. These events converge on RhoGTPases-dependent cytoskeletal remodeling and transcriptional reprogramming associated with EMT. CDH1 = E-cadherin; VIM = Vimentin; FN1 = Fibronectin 1. Created in BioRender. Piperigkou, Z. (2026) https://BioRender.com/hd39kos (accessed on 20 May 2026).

This multilayered transcriptional, post-transcriptional and translational regulation enables rapid adaptation of sCLU levels in response to cellular stress, such as oxidative damage, inflammation, and therapeutic exposure, promoting sCLU upregulation as part of a cytoprotective adaptive response [57]. Functionally, sCLU is best known as the most relevant extracellular molecular chaperone that binds misfolded proteins to prevent their aggregation and facilitate their clearance through receptor-mediated uptake [20]. This process is mainly mediated by LRP family receptors and cell-surface HSPGs [19,21]. Among these, LRP1 has emerged as a key functional receptor, coupling the endocytic clearance of sCLU-associated complexes with the activation of downstream signaling pathways that regulate cell survival and migration [23] (Figure 3).

Beyond its role in proteostasis, sCLU has been implicated in a wide range of biological processes, including lipid transport, apoptosis regulation, and tissue remodeling. Altered CLU expression has been reported in multiple malignancies, including breast [58], pancreatic [59], prostate [60], and colorectal cancer [61], where sCLU has been associated with the activation of pro-survival signaling pathways, tumor progression, metastatic dissemination, and therapy resistance. However, the role of CLU in tumorigenesis remains complex and highly context-dependent, as accumulating evidence indicates that its biological functions vary according to tumor stage, cellular localization, and microenvironmental conditions [62,63,64].

Indeed, although sCLU is frequently upregulated in advanced tumors and is generally referred to as a tumor-promoting, stress-adaptive factor, studies in CLU-deficient mice have consistently shown increased susceptibility to tumor initiation in models of prostate, brain, and skin carcinogenesis [64,65,66]. These apparently opposing observations can be reconciled by considering the fundamentally cytoprotective nature of CLU in both normal and transformed cells. In early phases of carcinogenesis, CLU exerts tissue-protective functions by limiting oxidative and proteotoxic stress, preserving extracellular homeostasis, and attenuating chronic inflammatory signaling, ultimately reducing the accumulation of DNA damage and the likelihood of malignant transformation. Consequently, complete CLU ablation may favor tumor initiation by impairing these homeostatic defense mechanisms. Conversely, once malignant transformation has occurred, cancer cells exploit the cytoprotective properties of sCLU to adapt to the hostile conditions of the TME, including hypoxia, nutrient deprivation, inflammatory stress, and therapeutic pressure. Under these conditions, persistent sCLU upregulation supports tumor cell survival, resistance to apoptosis, metastatic competence, and resistance to anticancer therapies [67].

The stage-dependent functions of CLU may also be influenced by the existence of distinct CLU isoforms characterized by different intracellular localization and biological activities [61]. Specifically, secretory/cytoplasmic CLU has largely been associated with pro-survival and tumor-promoting effects, whereas nuclear CLU has been linked to pro-apoptotic and growth-suppressive functions [68,69]. Together, these observations support the notion that the divergent roles of CLU in cancer are not contradictory but rather reflect the strong dependence of CLU biology on tumor stage, cellular context, stress conditions, and protein localization.

sCLU has been identified as a key mediator of EMT and tumor progression, acting through ERK signaling and Slug-dependent repression of epithelial markers (E-cadherin, encoded by CDH1) and upregulation of mesenchymal ones (fibronectin, FN1) [70]. Notably, sCLU is strongly upregulated downstream of TGF-β signaling during EMT in multiple cancer cell lines and represents one of the most responsive genes throughout this process [71]. Furthermore, sCLU promotes resistance to TNFα-induced cell death in breast cancer (BC) cells via NF-κB activation and subsequent upregulation of Bcl-2, thereby supporting tumor development and metastatic potential [72].

sCLU also plays a multifaceted role in the regulation of MMPs, positioning it as an important modulator of ECM turnover and tissue homeostasis, particularly at mucosal and epithelial surfaces. Mechanistically, sCLU can directly interact with several MMPs through its extracellular chaperone activity. By binding partially unfolded or stress-activated forms of these proteases, sCLU prevents their aberrant aggregation and can limit their proteolytic activity. In the case of MMP9, sCLU has been shown to form stable complexes that reduce enzymatic activity and protect key structural proteins such as tight junction components and basement membrane constituents from excessive degradation [73]. This inhibitory effect contributes to the preservation of epithelial barrier integrity under inflammatory or oxidative stress conditions. In parallel, sCLU may also regulate MMP expression at the transcriptional level in a context-dependent manner. In certain cell types, including immune and epithelial cells, sCLU has been reported to activate signaling pathways such as ERK1/2 and PI3K/Akt, leading to NF-κB-dependent upregulation of MMP9 [74,75]. In triple-negative breast cancer (TNBC) models, CLU silencing significantly impairs cell migration, invasion, tumor growth, and metastatic dissemination, in association with decreased ERK signaling and MMP9 protein levels [76,77]. This apparent duality, namely direct inhibition of MMP activity versus indirect stimulation of MMP expression, suggests that sCLU functions as a fine tuner of proteolytic balance rather than a simple inhibitor, adapting its role according to the microenvironment and the phase of tissue response (i.e., acute injury versus remodeling).

Overall, sCLU emerges as a multifunctional extracellular chaperone whose tightly regulated expression and ability to interface with proteolytic systems, such as MMPs, positions it at the crossroads of proteostasis, inflammation, and tissue remodeling. In line with this, the ability of sCLU to bind multiple extracellular partners suggests a broader involvement in organizing protein complexes at the cell surface, linking extracellular proteostasis to intracellular signaling. Through its interaction with LRP1 and other membrane-associated proteins, sCLU may promote the spatial organization of proteases and adhesion receptors at the plasma membrane, thereby modulating their activity.

## 4. Clusterin Guides an LRP1/MT1-MMP/CD44 Signaling Network Driving Tumor Cell Invasion

The accumulated evidence outlined above makes it plausible to suggest that sCLU may guide an LRP1/MT1-MMP/CD44/HSPGs signaling network driving tumor cell invasion (Figure 3). The binding of sCLU to LRP1 may act as a nucleation event for the organization of membrane-associated molecules. Indeed, sCLU may promote the interaction of LRP1 with proteases and adhesion receptors at the plasma membrane, thereby regulating their distribution and functional interplay. This spatial organization is expected to facilitate coordinated membrane trafficking and receptor turnover, further contributing to the dynamic regulation of receptor availability at the cell surface.

In this context, key components such as MT1-MMP and CD44 are likely to be recruited, enhancing localized ECM degradation, modulating adhesion dynamics, thereby acting as a signaling network that promotes MAPK and Akt activation, cytoskeletal remodeling, and cell motility. Consistent with these observations, proteomic analyses performed in TNBC cells revealed that CLU silencing resulted in widespread alterations in proteins implicated in cell adhesion, cytoskeletal organization, and ECM dynamics, supporting a role for sCLU in integrating ECM-derived cues with membrane receptor-mediated signaling and coordinating downstream structural and signaling networks [78].

These events also involve the modulation of Rho family GTPases, including RhoA, Rac1, and Cdc42, which coordinate membrane protrusion, contractility, and directional migration [79]. Notably, we found that CLU expression abrogation in TNBC cells is accompanied by the reduction intracellular RhoA-GTP levels [78], yielding the impaired activation of RhoA-dependent pathways that regulate actin cytoskeleton architecture. In line with this view, CD44 has been shown to engage a downstream tPA/LRP1/NF-κB signaling axis that promotes lamellipodia outgrowth and cell migration in BC cells [80]. This further supports a role for LRP1 as a key co-receptor within pro-migratory pathways, consistent with its proposed function in the sCLU-driven network described here.

Beyond the regulation of pro-migratory signaling, this membrane-associated network may also promote full or hybrid EMT-associated phenotypes/reprogramming. Notably, sCLU has been shown to interact with extracellular HSP90α to enhance LRP1 mediated Akt, ERK, and NF-κB signaling, thereby synergistically promoting EMT [23]. Similarly, the co-localization of CD44 and MT1-MMP at the leading edge has been demonstrated in cells undergoing EMT. Moreover, CD44 overexpression induces activation of the EGFR/PI3K/Akt signaling pathway while disrupting the membrane-associated E-cadherin-β-catenin complex, ultimately leading to β-catenin nuclear translocation and transcriptional activation of mesenchymal markers and invasion-related genes [81].

Collectively, available evidence may support a model in which sCLU drives the interaction of LRP1 with cell surface components contributing to proteolysis, receptor trafficking, and signaling to coordinate cytoskeletal remodeling and EMT programs that sustain tumor cell invasion. These insights also suggest that disrupting the coordinated interactions within this network may represent a promising strategy to interfere with tumor progression.

## 5. Syndecans as Possible Indirect Modulators of CLU/LRP1/MT1-MMP/CD44 Network in Tumor Cell Migration

As already mentioned, SDCs comprise multifunctional cell surface HSPGs, which hold central roles in cancer progression [82]. Of note, they are referred to regulate tumor cell proliferation, apoptosis, migration, invasion, angiogenesis, inflammatory signaling within the TME, as well as the maintenance of cancer stem cell properties associated with recurrence and therapeutic resistance [11]. Notably, their functions are highly context-dependent, varying with tumor type, stage, and cellular origin, and they often exert dual or even opposing effects depending on their localization and interaction partners [10,13].

In the context of cancer cell migration and invasion, SDCs act as critical modulators of cell–ECM interactions and associated signaling pathways [13]. Particularly, SDC1 regulates cell adhesion, where its loss is often associated with increased motility and invasiveness, while its extracellular domain can also participate in signaling complexes with integrins to promote cytoskeletal reorganization [13,83,84]. SDC2 has also been implicated in enhanced migratory behavior, partly through EMT-related signaling pathways [85]. Importantly, SDC4 functions as a key player in coordinating focal adhesion dynamics and actin cytoskeletal remodeling through signaling axes involving Rac1, protein kinase C alpha (PKCα), and focal adhesion kinase (FAK), thereby facilitating the formation of membrane protrusions required for efficient cell movement [27]. In contrast, SDC3 functional properties in cancer progression remains insufficiently explored; however, recent studies have demonstrated its role in BC cell migration [10].

Within this framework, the proposed CLU-associated LRP1/MT1-MMP/CD44 network driving tumor cell migration and invasion raised the possibility that HSPGs, particularly SDCs, may function as indirect modulators of this network (Figure 3). LRP1 acts as a multifunctional receptor involved in cell signaling and adhesion through interactions with various membrane-associated partners, including HSPGs such as SDC1 and GPC3, thereby influencing ligand availability and downstream signaling [27,45]. In particular, STRING-based PPI analyses revealed co-expression associations between LRP1 and SDC1, SDC2, SDC4, GPC1, GPC3, GPC4, and GPC6, as well as an experimentally validated interaction with HSPG2/perlecan (Figure 1B). STRING analysis further identified both co-expression and experimentally validated interactions among LRP1 and integrin β_1_, a key subunit shared by multiple integrin heterodimers (Figure 1C). It is already known that LRP1 interacts with several integrin heterodimers, including α_M_β_1_, α_M_β_2_, α_V_β_3_, and α_V_β_5_, as well as subunits α_M_, α_L_, β_1_, and β_2_, thereby regulating integrin availability/activity and, therefore, cellular adhesion/migration [4,86]. Evidence in the literature supports that LRP1–integrin interactions can modulate both integrin surface availability and functional activity [86]. In addition, integrin activation has been shown to be regulated by SDC-1 [87], while SDC-4 contributes to the dynamic assembly and disassembly of integrin-containing complexes at focal adhesion sites [88]. Specific SDC–integrin pairs, such as SDC4-α_5_β_1_, SDC1-α_V_β_3_/α_V_β_5_, and indirect SDC2-β_1_ are particularly important for adhesion to ECM proteins (fibronectin and vitronectin, respectively), and for subsequent cancer cell spreading and migration [11,26,87,89]. Given the central role of HSPGs/integrins in focal adhesion formation and cell motility [26], a coordinated functional interplay between LRP1, HSPGs, and integrins may represent an indirect regulatory network in CLU-mediated cancer cell migration.

The localization and functional role of MT1-MMP within migratory and invasive cellular protrusions has been already introduced. Extending these, MT1-MMP plays a central role during invadopodia maturation by coordinating the trafficking and cell surface presentation of matrix-degrading enzymes, together with SDC1. This coordinated delivery facilitates localized ECM degradation and contributes to basement membrane disruption [3]. Importantly, the co-expression of MT1-MMP with SDC1 (also shown in Figure 1B) suggests that the former cleaves the ectodomain of SDC1, leading to its proteolytic shedding from the cell surface. Of note, in the same study, shed SDC1 ectodomain was shown to enhance fibrosarcoma cell migration on collagen-rich substrates, indicating a functional role in modulating cell–ECM interactions and promoting cell motility [90]. Collectively, these data further support the concept that CLU-associated signaling may modulate the proposed network, thereby enhancing SDC1 ectodomain shedding and coordinating tumor cell migration and invasion.

To our knowledge, limited evidence is currently available regarding the direct interaction of CD44 with integrins and the downstream signaling pathways involved in cancer initiation [91]. However, STRING-derived co-expression analyses indicate associations between CD44 and multiple integrin subunits (i.e., ITGAV, ITGAL, ITGAM, ITGB2, ITGB3, and ITGB5), along with an experimentally validated interaction with ITGB1, suggesting a potential interplay within the sCLU-proposed model (Figure 1C). Additionally, co-expression of SDC1 and CD44 (as well as STRING-based CD44/SDC4 co-expression, Figure 1C) suggests a potential functional association of the two, which may further contribute to the regulation of cell adhesion and signaling pathways implicated in cancer progression [92]. Given the established role of CD44 in cancer stem cell phenotypes and migration, it is plausible that the aforementioned interactions may influence cancer (stem) cell motility, although direct mechanistic evidence remains unexplored.

## 6. Therapeutic Targeting and Clinical Relevance of the sCLU/LRP1-Driven Signaling Network

**Figure 4:**
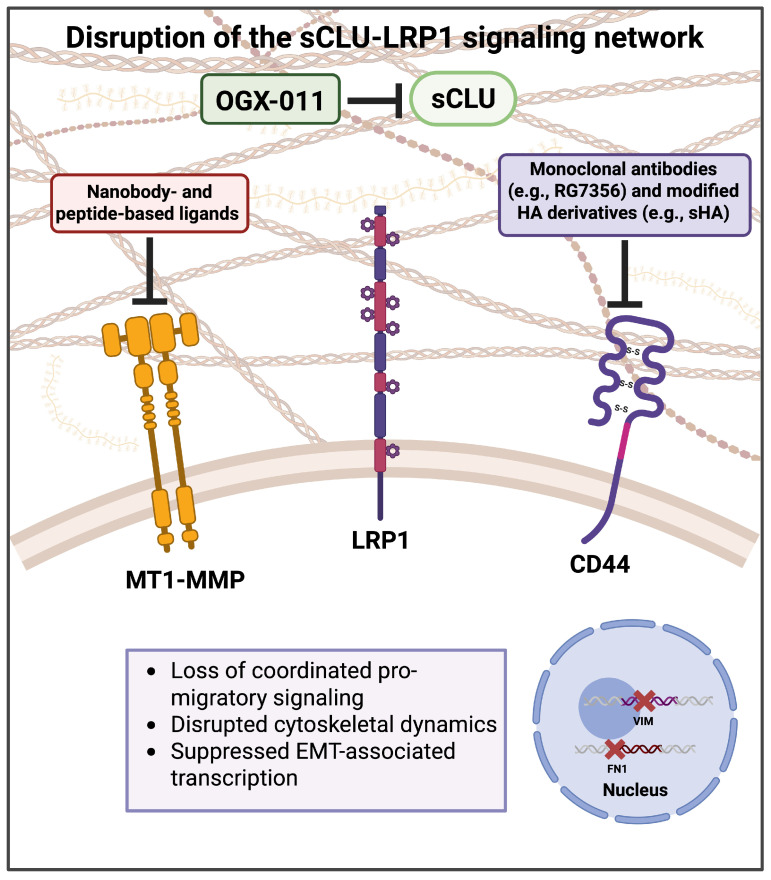
Disruption of the sCLU/LRP1-membrane associated network by targeted inhibition. Pharmacological inhibition of sCLU (i.e., OGX-011) prevents membrane cluster formation. The simultaneous targeting of key components of the network (MT1-MMP and CD44) results in loss of coordinated receptor activity and intracellular signaling, impaired cytoskeletal dynamics, and suppression of EMT-associated transcription, ultimately limiting tumor cell migration and invasion. VIM = Vimentin; FN1 = Fibronectin 1. Created in BioRender. Piperigkou, Z. (2026) https://BioRender.com/1atzxhh (accessed on 20 May 2026).

Beyond its mechanistic relevance, the identification of an sCLU-LRP1-associated signaling network may provide a conceptual framework for the development of therapeutic strategies aimed at disrupting coordinated pro-invasive programs in cancer (Figure 4).

Targeting sCLU with antisense oligonucleotides such as OGX-011 has exhibited high efficacy in hampering tumor growth, invasion, and metastatic dissemination across multiple preclinical cancer models [93,94]. In clinical settings, the therapeutic potential of CLU inhibition was tested in combination with standard chemotherapeutic treatments. Phase I and Phase II clinical studies demonstrated that OGX-011 is well tolerated and effectively reduces sCLU serum levels, leading to encouraging changes in biomarkers used as surrogate endpoints of exploratory efficacy [95,96,97]. However, despite these promising findings, two large phase III clinical trials failed to demonstrate a significant survival benefit in patients with metastatic castration-resistant prostate cancer [98,99]. These discrepancies highlight the limitations of selectively targeting sCLU within a broader and highly interconnected pro-invasive signaling network. In line with this, the sCLU-driven network described here provides a potential explanation for these outcomes, as sCLU likely acts within a broader membrane-associated signaling hub integrating receptor trafficking, proteolysis, and activation of intracellular effector molecules. Therefore, targeting CLU alone may be insufficient to fully disrupt the coordinated pro-invasive programs that sustain tumor progression, supporting the need for combinatorial strategies.

In this context, additional molecular components of the proposed pro-tumorigenic network represent attractive therapeutic targets. Although LRP1 plays a central role in mediating sCLU-driven clustering and signal integration, its wide-ranging expression across different tissues and cell types, together with its ability to bind a broad spectrum of ligands and participate in multiple biological processes, has limited its direct therapeutic targeting [100]. This, in turn, supports the exploration of alternative, more druggable nodes within the network.

A growing body of evidence supports the development of CD44-targeted strategies, including monoclonal antibodies, nanobody- and peptide-based ligands, as well as hyaluronic acid (HA)-based drug delivery systems, which aim to selectively interfere with CD44-mediated signaling [101]. Notably, the humanized monoclonal antibody RG7356 has been investigated in a phase I trial in patients with metastatic or locally advanced CD44-expressing solid malignancies [102]. While this study demonstrated an acceptable safety profile and evidence of biological activity, clinical efficacy was limited, suggesting that combination strategies may be required to improve therapeutic outcomes. Recognizing HA as a major ligand of CD44, modified HA derivatives such as sulfated HA (sHA) have emerged as promising anticancer agents that interfere with CD44 availability and signaling, thereby attenuating tumor progression, including cell migration [24,103]. Notably, sHA has been shown to attenuate prostate cancer (PC) cell migration, inhibiting PI3K/Akt signaling, and inducing transcriptional downregulation of CD44, while also reducing tumor growth and angiogenesis in PC mouse models [104]. Similarly, sHA suppresses cell proliferation, migration, and invasion, but also downregulates CD44, thereby inhibiting CD44/PI3K complex formation and impairing tumorigenic signaling pathways in bladder cancer xenografts [105].

MT1-MMP has been extensively explored as a therapeutic target. Early attempts to inhibit MT1-MMP activity relied on broad-spectrum MMP inhibitors, which target the catalytic Zn^2+^-binding site. Although these compounds initially showed promising anti-tumoral effects in preclinical models, they largely failed in clinical settings due to poor selectivity, dose-limiting toxicity, and off-target inhibition of physiologically important MMPs involved in tissue remodeling, wound healing, and immune regulation. In addition, functional redundancy among MMP family members and the lack of appropriate patient stratification strategies likely further contributed to the limited clinical efficacy of these approaches. Recent strategies have focused on selectively targeting MT1-MMP through nanobodies and peptide-based ligands that interfere with substrate recognition or protein–protein interactions [106]. Although these approaches have shown promising anti-invasive and anti-angiogenic effects in preclinical models, their translation into clinical benefit has remained limited. As for CD44, the modest success of MT1-MMP-targeted interventions may reflect the fact that MT1-MMP does not act as an isolated protease, but rather as part of membrane-associated multiprotein complexes involved in different processes. Within the signaling network proposed here, MT1-MMP is functionally coupled to receptors such as CD44 and LRP1, suggesting that its inhibition alone may be insufficient to disrupt the coordinated pro-invasive programs that drive tumor progression.

Although these compounds initially showed promising anti-tumoral effects in preclinical models, they largely failed in clinical settings due to poor selectivity, dose-limiting toxicities, and off-target inhibition of physiologically important MMPs involved in tissue remodeling, wound healing, and immune regulation. In addition, functional redundancy among MMP family members and the lack of appropriate patient stratification strategies likely further contributed to the limited clinical efficacy of these approaches.

## 7. Limitations and Future Perspectives

Despite the integrative framework proposed here, few limitations should be acknowledged. First, the model, although supported from accumulated literature data, it remains as a proposed hypothetical network where several points have to be validated experimentally. While supported by published evidence and bioinformatic interaction network analyses, the proposed membrane-associated interactions among sCLU, LRP1, MT1-MMP, CD44, and related ECM molecules have not been demonstrated as a coordinated dynamic system in space and time. Importantly, the present framework does not imply the existence of a stable or simultaneous multiprotein complex, as these interactions may occur sequentially, transiently, or in a context-dependent manner based on extracellular cues and TME conditions. Another limitation derives from the intrinsic redundancy of membrane-associated signaling and proteolytic networks, where compensatory mechanisms involving alternative receptors, proteases, or HSPGs may partially substitute for individual pathway components.

Future studies will be required to experimentally validate the proposed framework and to clarify the spatial and temporal organization of these membrane-associated interactions in tumor cells. In this context, complementary approaches such as co-immunoprecipitation, proximity ligation assays, and super-resolution microscopy could help determine whether sCLU, LRP1, MT1-MMP, CD44, and related ECM-associated molecules dynamically assemble into functionally coordinated membrane-associated platforms. In parallel, live-cell imaging may provide important insights into the spatiotemporal dynamics, trafficking behavior, and transient membrane organization of these components in real time. Moreover, the implementation of advanced experimental models, such as 3D cell cultures, will be essential to determine the biological relevance of this framework within the complex TME. Collectively, these approaches may help clarify the functional and translational relevance of this proposed membrane-associated framework in breast cancer progression.

## 8. Conclusions

Literature data and STRING interaction analyses support the idea that sCLU may function as an upstream regulator of a membrane-associated signaling network centered on LRP1, integrating proteolysis, endocytosis, and intracellular pathways. By engaging LRP1, sCLU may promote the assembly of MT1-MMP and CD44, thereby enhancing localized ECM degradation and modulating adhesion dynamics. In parallel, the cytoplasmic domain of LRP1 contributes to intracellular signaling pathways that support cytoskeletal remodeling and cell motility. HSPGs may further contribute to this network since they are known to interact with LRP1 and regulate the availability of TGF-β, linking in this way the ECM architecture to EMT signaling and may further modulate cytoskeletal dynamics through Rho GTPase-dependent pathways. Within this framework, CD44 is widely recognized as a marker of cancer stemness in several tumor types, linking this promigratory axis to stemness-associated traits and tumor aggressiveness. Consistently, increased expression of both CLU and LRP1 has been associated with poor prognosis, highlighting the clinical relevance of this hypothetical model. Therefore, targeting the sCLU/LRP1/MT1-MMP/CD44 network at multiple levels may represent a potential therapeutic strategy to limit tumor cell plasticity and metastatic dissemination in aggressive cancers.

## Figures and Tables

**Table 1:** Localization and biological functions of key membrane-associated network (sCLU, LRP1, MT1-MMP, CD44, SDCs, GPCs, and perlecan) components.

Protein	Abbreviation	Localization	Biological Functions	Reference
Secreted clusterin	sCLU	Extracellular space	Extracellular chaperone; protein clustering; stress response	[18,20]
Low-density lipoprotein receptor-related protein 1	LRP1	Cell membrane *	Endocytosis; intracellular signaling	[4]
CD44	CD44	Cell membrane *	Cell–ECM adhesion; scaffold for MMPs	[24]
Membrane Type 1 Matrix Metalloproteinase	MT1-MMP (MMP14)	Cell membrane	ECM degradation; receptor shedding; pro-MMP2 activation	[3]
Syndecans	SDC1	Cell membrane *	Cell–ECM adhesion; growth factor signaling; integrin-mediated signaling; shed SDC1 signaling	[13,25]
	SDC2	Cell membrane *	Cell–ECM adhesion; growth factor signaling; integrin-mediated signaling; shed SDC2 signaling	[26]
	SDC4	Cell membrane *	Focal adhesion formation; cytoskeletal remodeling; growth factor signaling; integrin-mediated signaling; shed SDC4 signaling	[27]
Glypicans	GPC1-6	Cell membrane *	Cell surface co-receptors; cell–matrix interactions.	[15]
Perlecan	HSPG2	Pericellular/basement membrane	ECM/basement membrane organization; cell adhesion; growth factor sequestration; integrin-mediated signaling	[16]

* Note: These cell surface molecules may also be detected in the extracellular space following shedding.

## Data Availability

No new data were created or analyzed in this study. Data sharing is not applicable to this article.

## Abbreviations

The following abbreviations are used in this manuscript:

ECM	Extracellular matrix
EMT	Epithelial to mesenchymal transition
TME	Tumor microenvironment
MT1-MMP	Membrane type 1 matrix metalloproteinase
LRP1	Low-density lipoprotein receptor-related protein 1
CLU	Clusterin
sCLU	Secreted clusterin
SDCs	Syndecans
GPCs	Glypicans
HSPG2	Perlecan
CD44	Cluster of differentiation 44
MMPs	Metalloproteinases
PGs	Proteoglycans
TIMP2	Tissue inhibitor of metalloproteinase 2
GFRs	Growth factor receptors
ER	Endoplasmic reticulum
BC	Breast cancer
TNBC	Triple-negative breast cancer
HA	Hyaluronic acid
sHA	Sulfated hyaluronic acid
PC	Prostate cancer
PPI	Protein–protein interaction
